# Constructing a global transcriptional regulatory landscape for early non-small cell lung cancer to identify hub genes and key pathways

**DOI:** 10.18632/aging.103475

**Published:** 2020-09-14

**Authors:** Jianlong Bu, Pinyi Zhang, Kaibin Zhu, Yubo Yan, Bowen Shi, Junfeng Wang, Shidong Xu

**Affiliations:** 1 Department of Thoracic Surgery, Harbin Medical University Cancer Hospital, Harbin 150081, Heilongjiang Province, China; 2 Department of Anesthesiology, Harbin Medical University Cancer Hospital, Harbin 150081, Heilongjiang Province, China

**Keywords:** non-small cell lung cancer, LUAD, LUSC, hub gene, tumor heterogeneity

## Abstract

This study aimed to investigate the potential pathogenesis of early non-small cell lung cancer (NSCLC), including lung adenocarcinoma (LUAD) and lung squamous cell carcinoma (LUSC), by constructing a global transcriptional regulatory landscape to identify hub genes and key pathways. A total of 1,206 differentially expressed genes (DEGs) in early NSCLC were identified compared to normal lung tissue samples in GSE33532 and GSE29013. DEGs-related protein-protein interaction networks (PPIs) were constructed based on the STRING database and were then modularly analyzed using the ClusterOne tool. The enrichment analysis revealed that multiple modules were significantly involved in pathways such as the TNF signaling pathway, PPAR signaling pathway and PI3K/AKt signaling pathway. Ten genes were identified as hub genes in the PPIs and also found up-regulated at protein level. The prognostic value of the hub genes and the ten hub gene set variation score varied according to the different pathological types of NSCLC, which suggested the ten hub gene expression patterns can reflect the heterogeneity of two types of NSCLC. In conclusion, by carrying out a series of in-depth analyses, hub genes and key pathways associated with early NSCLC were identified by a global transcriptional regulatory landscape.

## INTRODUCTION

Non-small cell lung (NSCLC) is one of the most common malignant tumors in the world, accounting for 80% of all lung cancers [1, 2]. Early (I-II stage) NSCLC has a good prognosis, with a 5-year relative overall survival rate of more than 60%, however, only 25% of patients with NSCLC are diagnosed at this stage [3]. Therefore, it is necessary to urgently determine the pathogenesis of early NSCLC and investigate the biomarkers and key pathways related to NSCLC for its early diagnosis and treatment.

Epidermal growth factor receptor (EGFR), anaplastic lymphoma kinase (ALK), ROS1 proto-oncogene receptor tyrosine kinase (ROS1), and serine/threonine-protein kinase B-Raf (BRAF) have proven to be genetic causes and effective therapeutic targets for selected patients with NSCLC [4–7]. These agents, however, are not suitable for a large proportion of those with NSCLC, and the associated effects are generally incomplete and temporary. Obviously, additional similar hub genes are required in this regard. Notably, most studies often focus on single pathological type of NSCLC as well as single or multiple genes, which may reveal a limited aspect of the pathogenesis of NSCLC. Furthermore, few reports mention the two most common pathological types of NSCLC from a global perspective.

Therefore, the present study explores the functional modules from differentially expressed gene-related protein-protein interaction networks (PPIs) in NSCLC from a global perspective. The pathways in which multiple functional modules are involved may serve as potential key pathways for early NSCLC. Potential hub genes were identified, and their aberrant expressions were validated. Their corresponding prognostic values were also explored in an independent data set.

## RESULTS

**Figure 1:**
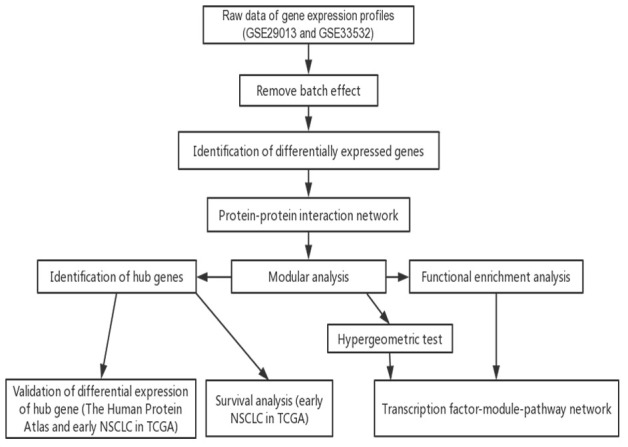
**low chart of this study.** TCGA, The Cancer Genome Atlas; NSCLC, non-small cell lung cancer.

In the present study, the DEGs among the early NSCLC tissue samples and normal lung tissue were used to construct the PPI networks. The key pathways of the early NSCLC samples were identified via modular and enrichment analyses, while the TFs that drive NSCLC were identified by the hypergeometric test. Accordingly, a global regulatory landscape related to early NSCLC was constructed. The differential expression of the hub genes was validated, and its prognostic value was explored in the early LUAD and LUSC datasets in TCGA (Figure 1).

### Atlas of expression imbalance in early NSCLC

**Figure 2:**
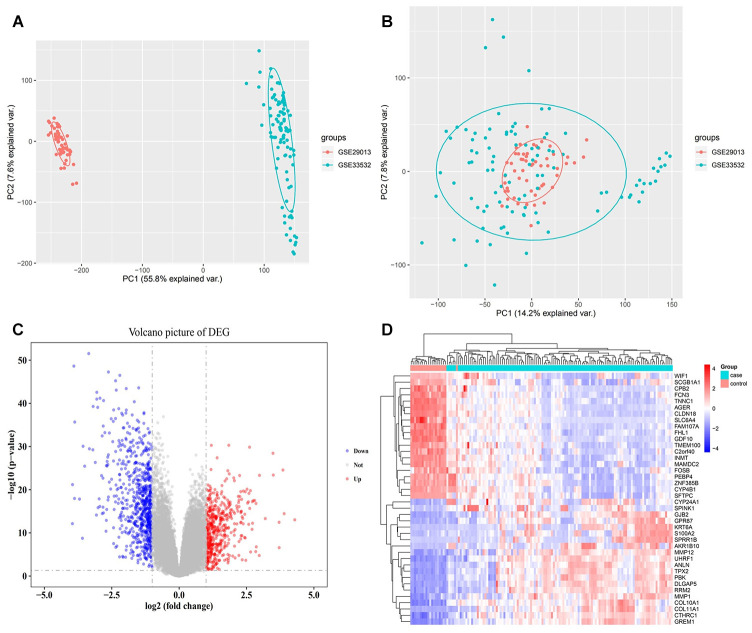
**Expression disorders of early NSCLC.** (**A**) PCA analysis before batch effect removal. (**B**) PCA analysis following batch effect removal. (**C**) Differentially expressed gene (DEG) volcano map. Red nodes represent upregulated genes, blue nodes represent downregulated genes, and gray nodes represent no differentially expressed genes. (**D**) Hierarchical clustering dendrograms of the expression patterns of differently expressed genes that distinguish between NSCLC and normal lung tissue.

The obtained PCA results demonstrated that the batch effect present in the two data sets were well removed (Figure 2A, 2B). A total of 1,206 DEGs (Figure 2C) were differentially expressed in early NSCLC compared to normal lung tissue samples, with 487 being significantly upregulated and 719 being significantly downregulated. Cluster analysis illustrated that the expression patterns of the corresponding DEGs could distinguish early NSCLC tissue samples from normal lung tissue samples (Figure 2D).

### NSCLC is the result of multiple functional modules

**Figure 3:**
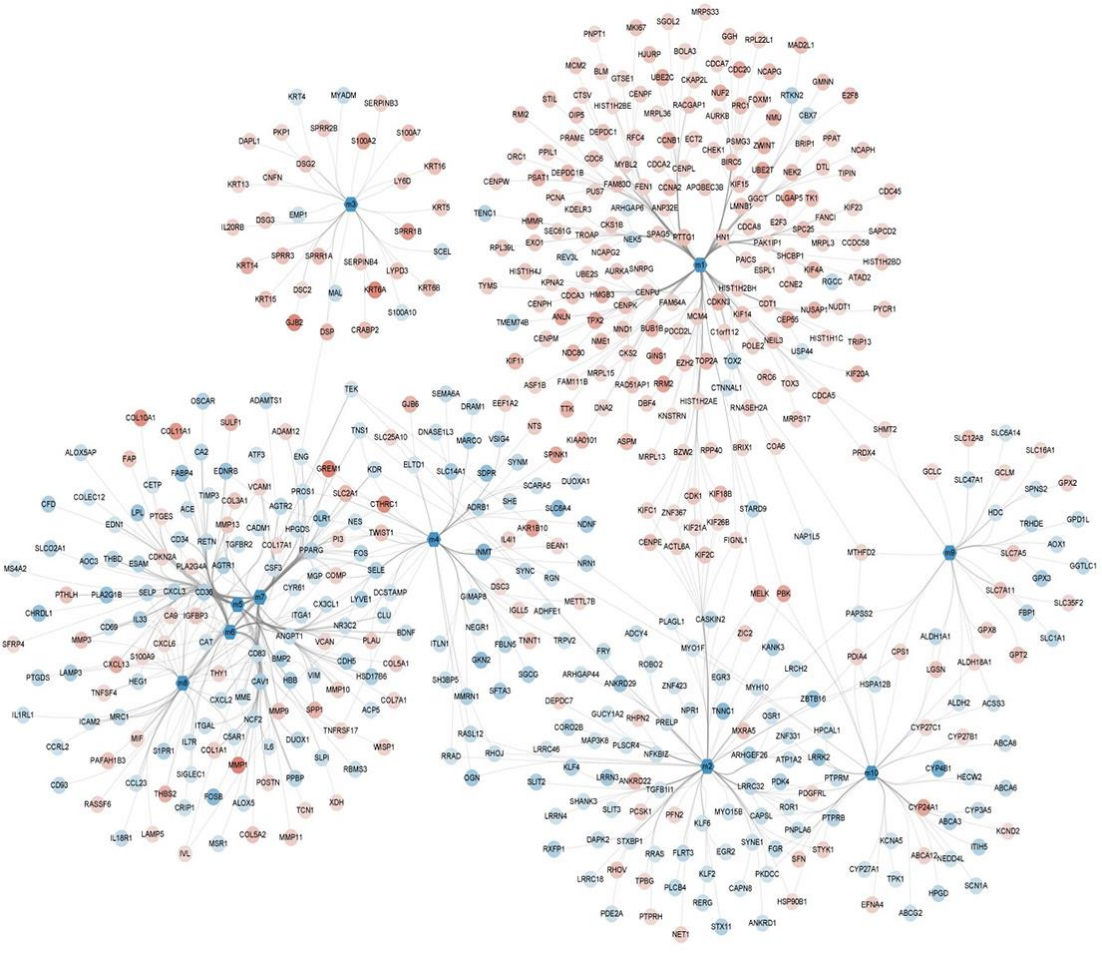
**Module network showing the modules and their gene members with color mapping logFC of their differential expressions.**

Here, 1,206 DEGs were mapped into the STRING database in order to construct a PPI network containing 1,206 nodes and 29,677 interaction pairs. Ten functional modules were identified comprised of 803 DEGs (Figure 3). Modularization helped to observe the complex interactions between these DEGs in regard to close protein interaction. To this effect, the occurrence of NSCLC was found to be the result of the combined action of multiple functional modules.

### Key pathways for early NSCLC

**Figure 4:**
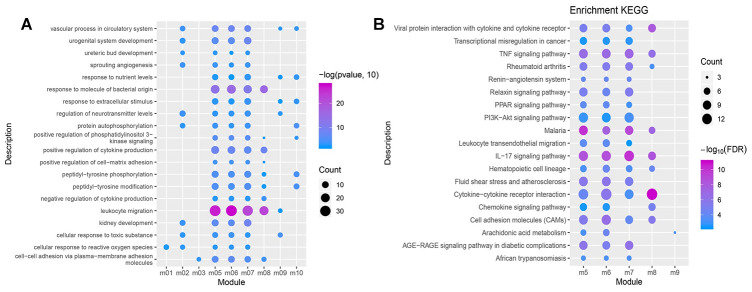
**GO function and KEGG pathway of the functional module.** (**A**) Biological processes having more than 3 functional modules are significantly enriched. (**B**) KEGG pathways having more than 2 functional modules were significantly involved.

The conducted enrichment analyses (GO and KEGG) suggest that these functional modules were significantly enriched in 4,467 biological processes (BPs), 317 cellular components (CCs), 685 molecular functions (MFs), and 120 KEGG pathways. The 20 biological processes (BP) containing over three functional modules involved inflammatory responses and immune functions (Figure 4A), which may serve as potential key BPs in early NSCLC. More than two functional modules were involved in 19 pathways, such as the cytokine-cytokine receptor interaction signaling pathway, IL-17 signaling pathway, TNF signaling pathway, PPAR signaling pathway and PI3K/AKt signaling pathway (Figure 4B), which may constitute potential key pathways of early NSCLC.

### Global regulation landscape of early NSCLC

**Figure 5:**
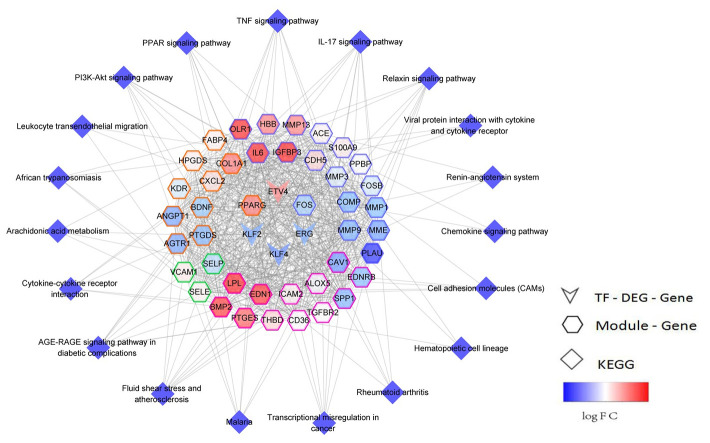
**TF-module-pathway comprehensive regulated network landscape of early NSCLC.** The network center is the transcription factor, while the color map of the gene node is logFC, and the color of the gene node side represents different modules.

Ninety-seven TFs that regulated the 10 functional modules were identified via hypergeometric test. Here, 6 TFs were found to be significantly dysregulated, and their target genes were significantly involved in the key pathways of early NSCLC. Subsequently, a TF-module-pathway network (Figure 5) was built to construct a novel globally-controlled landscape map of NSCLC.

### Hub gene is a potential biomarker for early NSCLC prognosis

**Figure 6:**
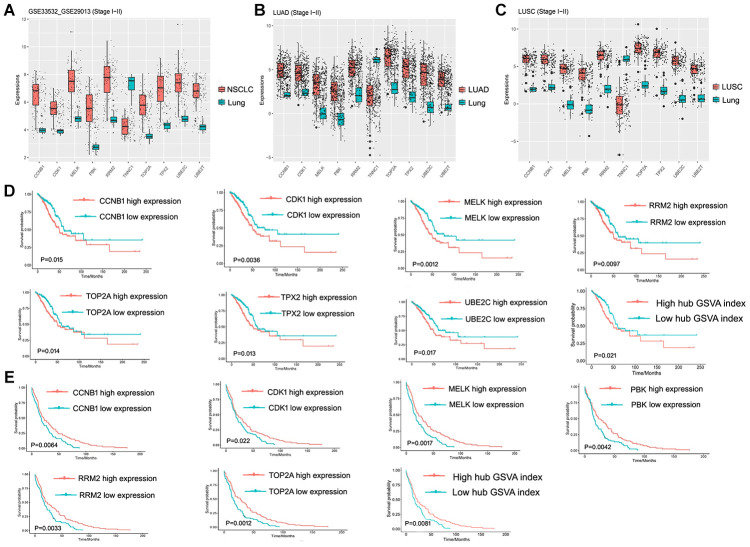
**Hub gene and its prognostic value. (A**) Expression of hub genes in GSE33532 and GSE29013. (**B**) Expression of hub genes in the early LUAD data set from TCGA. (**C**) Expression of hub genes in the early LUSC data set from TCGA. (**D**) Hub Genes and the hub GSVA index associated with prognosis in the early LUAD data set from TCGA. (**E**) Hub Genes and the hub GSVA index associated with prognosis in the early LUSC data set from TCGA. LUAD, lung adenocarcinoma; LUSC, Lung squamous cell carcinoma; GSVA, gene set variation analysis; TCGA, The Cancer Genome Atlas.

**Figure 7:**
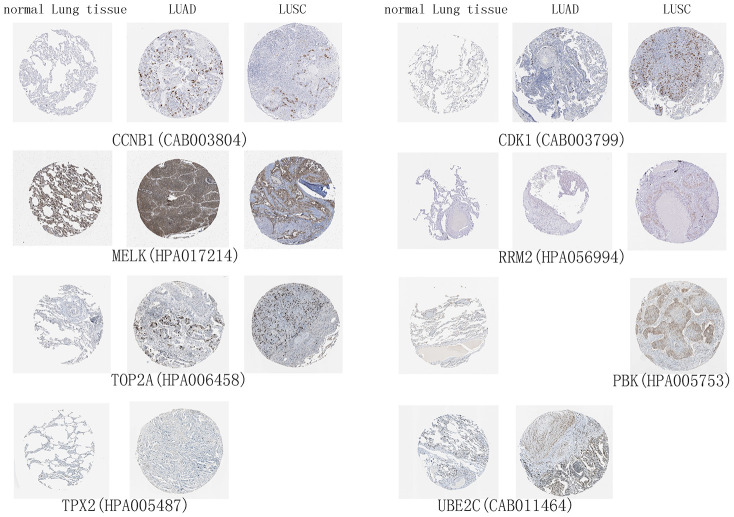
**High expression of hub genes in immunohistochemistry.** Normal Lung tissue samples are on the left, lung adenocarcinoma (LUAD) samples are in the middle, and lung squamous cell carcinoma (LUSC) samples are on the right.

The 10 hub genes were observed to be UBE2T, PBK, MELK, TNNC1, CCNB1, RRM2, CDK1, TOP2A, TPPX2 and UBE2C, which had top W values (Table 1). The differential expression of these 10 hub genes (Figure 6A) was verified in the LUAD (Figure 6B) and LUSC (Figure 6C) data sets from TCGA. A high expression of CCNB1, MELK, RRM2, CDK1, TOP2A, TPX2, and UBE2C in LUAD patients was found to be associated with a poor prognosis (Figure 6D), while a high expression of CCNB1, MELK, RRM2, CDK1, TOP2A, and PBK genes was observed to be associated with a good prognosis in LUSC patients (Figure 6E). The prognostic value of the hub genes differed according to the various pathological types of NSCLC. Moreover, the high hub GSVA index was associated with a better prognosis in LUSC, which contrasted with that of LUAD. This indicates that significant heterogeneity in LUAD and LUSC is present. In addition, some hub genes were also observed to be highly expressed in LUAD and LUSC compared to normal lung tissue at the protein level (Figure 7). The genes UBE2T, TNNC1, and TPPX2 were not available in The Human Protein Atlas.

**Table 1:** Hub genes.

**Symbol**	**Module**	**Degree**	**Log 2 fold change**	**Adjust P value**	**Weight**	**Rank**
UBE2T	m1	140	2.612245066	3.28E-28	10917.23979	1
PBK	m1	227	2.753660067	1.17E-16	10912.44999	2
PBK	m10	227	2.753660067	1.17E-16	10912.44999	2
PBK	m2	227	2.753660067	1.17E-16	10912.44999	2
MELK	m10	225	2.618661314	5.17E-15	9233.525148	3
MELK	m1	225	2.618661314	5.17E-15	9233.525148	3
MELK	m2	225	2.618661314	5.17E-15	9233.525148	3
TNNC1	m2	65	-3.056720526	2.03E-38	8143.511081	4
CCNB1	m1	156	2.517999686	4.14E-19	7904.540951	5
RRM2	m1	140	2.802407994	1.08E-16	6865.254987	6
TPX2	m1	129	2.644431171	8.34E-19	6749.700127	7
UBE2C	m1	130	2.589317511	3.24E-18	6445.158204	8
CDK1	m1	210	1.653412806	1.89E-17	6362.173919	9
CDK1	m2	210	1.653412806	1.89E-17	6362.173919	9
TOP2A	m1	157	2.193051181	1.42E-17	6355.448547	10

## DISCUSSION

Detecting early NSCLC is critical due to its greater chance for survival [8]. Therefore, this study attempted to determine the hub genes and key pathways of early NSCLC from a global perspective in regard to its pathogenesis. In this regard, PPI networks and a modular analysis based on DEGs in early NSCLC samples were conducted. Each module may represent the potential pathogenesis of NSCLC, and NSCLC was determined to be the result of the combined action of multiple functional modules. These functional modules are significantly involved in the inflammatory response as well as biological immune functions, suggesting that the development of early NSCLS is closely related to the immune system, while immune evasion is a mechanism of tumorigenesis [9]. Multiple functional modules were involved in the cytokine-cytokine receptor interaction signaling pathway, IL-17 signaling pathway, TNF signaling pathway, PPAR signaling pathway and PI3K/AKt signaling pathway, which may serve as potential pathways in the promotion of early NSCLC. The IL-7 signaling pathway promotes the pathogenesis of NSCLC [10], and activation of the TNF signaling pathway via inflammatory response may result in a poor prognosis of NSCLC [11]. It has also been verified that the PI3K/AKt signaling pathways are involved in the regulation of apoptosis in NSCLC cells, suggesting that the PI3K/AKt signaling pathway is associated with NSCLC [12]. In addition, this study’s proposed TF-module-pathway network may provide a reference for the additional research pertaining to the pathogenesis of NSCLC.

This study identified 10 hub genes: UBE2T, PBK, MELK, TNNC1, CCNB1, RRM2, CDK1, TOP2A, TPPX2, and UBE2C. Unsurprisingly, previous studies have found that some of these genes were associated with NSCLCs. The ubiquitin-binding enzyme E2C (UBE2C) gene is amplified in approximately 7% of NSCLC patients, suggesting its role in the pathophysiology of NSCL [13]. The CDK1 and MELK proliferation-related genes may serve as biomarkers of NSCLC immune checkpoint inhibitor therapy [14]. A high expression of RRM2 is a poor prognostic factor for LUAD and is a biomarker for the LUAD potential prediction of metastasis and prognosis [15], though it may be favorable for LUSC, according to the corresponding obtained results. A similar phenomenon was evident in CDK1, MELK, and UBE2C, which was unreported, suggesting that LUAD and LUSC have significant heterogeneity and different potential therapeutic targets. The prognostic value of the hub genes varied according to the different pathological types of NSCLC. In conjunction with previous studies, the 10 hub genes obtained in this study may help to elucidate the molecular mechanism of NSCLC.

Although the present study proposed potential hub genes and key pathways for early NSCLC, it has several limitations. First, the gene modules were mined based on the PPI networks from the STRING database, where some proteins were based on prediction rather than molecular experimentation. Thus, the molecular mechanism of these key pathways and hub genes require further molecular investigation. Second, less annotated pathways may be lost when filtering modules using the PPI network. Weighted correlation network analysis (WGCNA) [16] is an alternative module mining method, which is mainly based on the correlation of genes.

## CONCLUSION

In conclusion, this study identified potential hub genes and related pathways of early NSCLC from a global perspective in order to provide a reference for the study of the pathogenesis of early NSCLC.

## MATERIALS AND METHODS

### Data collection and processing

In the present study, two NSCLC gene expression profile datasets were downloaded from the Gene Expression Omnibus (GEO, https://www.ncbi.nlm.nih.gov/geo/) database, GSE33532 and GSE29013. The data set of GSE33532 includes 80 early NSCLC tissue samples (40 adenocarcinomas, 16 squamous cell carcinomas, and 24 NSCLCs of mixed type) and 20 normal lung tissue samples. The GSE29013 data set contains a total of 55 NSCLC samples including 38 early stage NSCLC samples (22 adenocarcinomas and 16 squamous cell carcinomas) and 17 advanced (III-IV stage) NSCLC samples. Both data sets were based on the GPL570 platform. The justRMA method in the affy package [17] was applied to normalize the raw data of the two data sets, and the sva package [18] removed the batch effect on the normalized data. After removing 17 advanced NSCLC samples, 118 NSCLC tissue samples and 20 normal lung tissue samples were utilized in the study. If one gene corresponded to multiple probes, the average expression value of these probes was considered to be the expression value of the gene. Principal component analysis (PCA) was also used to evaluate removing batch effects.

### Differentially expressed gene (DEG) analysis and bidirectional hierarchical clustering

The DEGs between early NSCLC and normal lung tissues were analyzed by the limma package [19] in R. Genes with |logFC| > 1 and P value adjusted by false discovery rate (FDR) < 0.05 were considered to be significant. Hierarchical clustering was performed using 20 of the most upregulated DEGs as well as 20 of the most downregulated DEGs using the pheatmap package (https://CRAN.R-project.org/package=pheatmap) in R.

### Construction of protein-protein interaction (PPI) networks and modular analysis

According to the STRING database (https://string-db.org/, [20]), PPI networks were constructed related to the DEGs. Network visualization was performed by Cytoscape [21] and the modules were analyzed (Minimum=30) by the ClusterONE plugin [22]. The DEGs-related PPI networks were organized into different functional modules.

### Enrichment analysis

The ClusterProfiler package in R was used [23] to perform Gene Ontology (GO) and Kyoto Encyclopedia of Genes and Genomes (KEGG) enrichment analysis for the functional modules. P adjusted by false discovery rate (FDR) < 0.05 was considered to be statistically significant.

### Module-related transcription factor (TF)

Based on the interaction of human TF and its target genes in the TRRUST v2 database (http://www.grnpedia.org/trrust/) [24], hypergeometric testing was applied to predict the TFs of potential regulatory functional modules. The hypergeometric test was performed using the igraph package (https://igraph.org/r/) in R. A P value of < 0.05 was considered to be statistically significant. As a result, a TF-module-pathway network was built.

### Differential expression validation, gene set variation analysis (GSVA) and survival analysis of hub gene set

After removing nodes not present in any of the modules within the PPI networks, the degree of each node was calculated, where the weight of a gene was W = -log10 (P value) x degree x |logFC|. The 10 genes with the top W values were considered to be hub genes. The early LUAD and LUSC data sets in The Cancer Genome Atlas (TCGA, https://www.cancer.gov/) were used to validate the differential expression of the hub genes, and the limma package's voom function was used to normalize the RNA-seq data for these two data sets. Additionally, the hub GSVA index was calculated for each sample using the GSVA package in R [25]. To explore the prognostic value of the expression of these 10 hub genes as well as the hub GSVA index in early NSCLC, the median was selected as the cutoff to divide early NSCLCs into high expression/index group and low expression/index group in TCGA data. The Kaplan-Meier survival curves of the two groups were compared using the Log-rank method, and a P value < 0.05 was considered to be statistically significant.

### Validation of the differential expression of hub genes at the protein level

The Human Protein Atlas (https://v15.proteinatlas.org/) [26] provides information on the tissue and cell distribution of all 24,000 human proteins, which was used to validate the differential expression of hub genes at the protein level in the present study.
